# Supplementary figures and images for: Phosphoregulation of HORMA domain protein HIM-3 promotes asymmetric synaptonemal complex disassembly in meiotic prophase in Caenorhabditis elegans
Source: PLoS Genet. 2020 Nov 11;16(11):e1008968. doi: 10.1371/journal.pgen.1008968 (PMC7717579; doi:10.1371/journal.pgen.1008968)

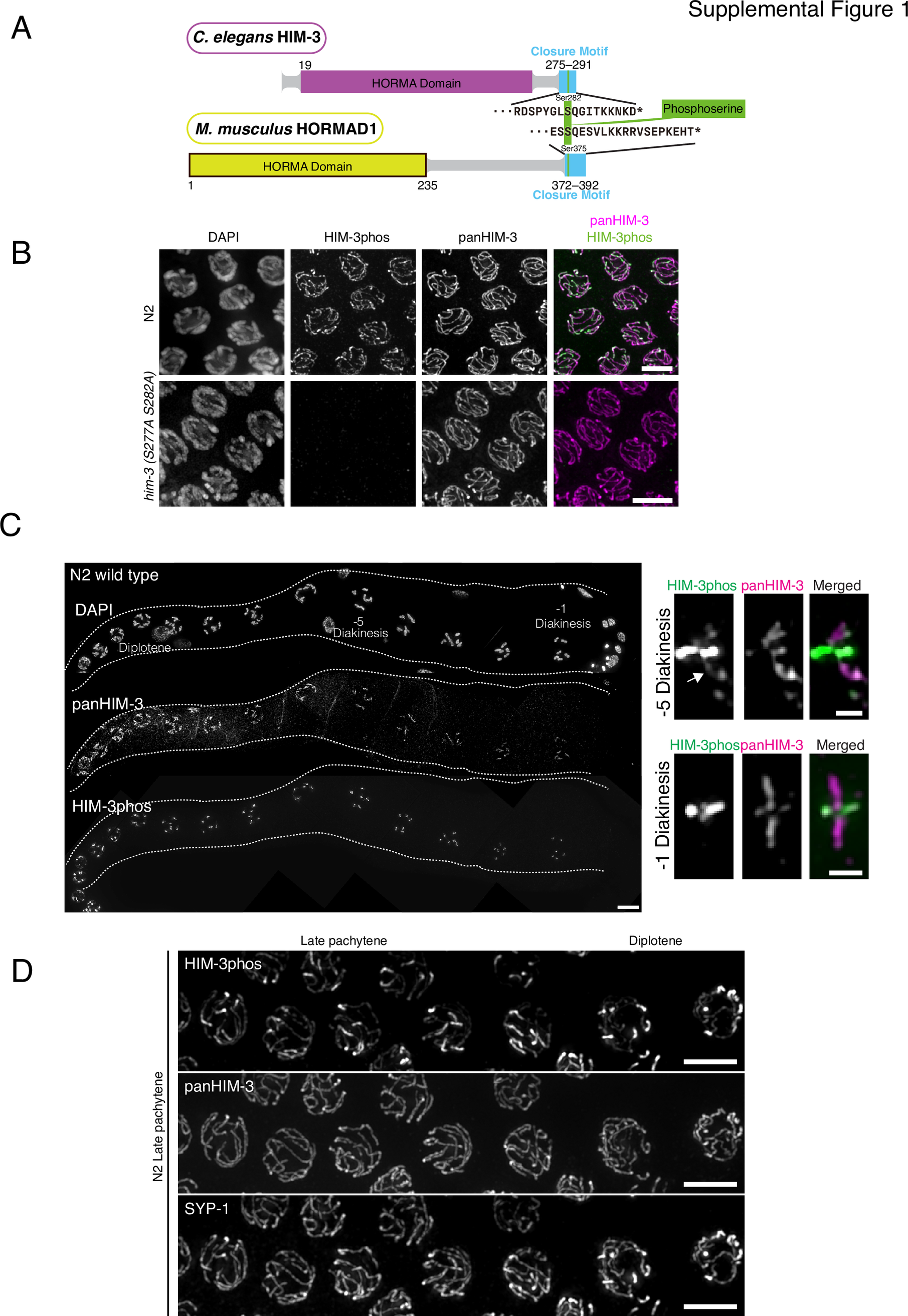

Supplement: S1 Fig — A, Diagrams of C. elegans HIM-3 (top) and mouse HORMAD1 (bottom), showing the positions of the closure motif and phosphoserine at the C-terminus. B, Antibodies specific to the phosphorylated closure motif stain the meiotic chromosome axis in wild-type (top, green in merged image) but not in S277A,S282A mutants (bottom), whereas pan-HIM-3 antibodies stain both (magenta in merged images), verifying the antibody’s specificity to the phosphoepitope. Scale bars, 5μm. C, Immunostaining of panHIM-3 and HIM-3phos in the N2 wild type gonad to show the progression of HIM-3phos enrichment to confinement. While HIM-3phos signals are mostly enriched on short arms from late pachytene, faint HIM-3phos staining, indicated by an arrow, is still detectable on long arms in early diakinesis and then disappears from long arms by -1 diakinesis. Scale bars, 5μm in the left panels and 1μm in the right panels. D. Immunostaining of HIM-3phos, panHIM-3 and panSYP-1 from late pachytene through diplotene to show temporal progression of HIM-3phos and panSYP-1 enrichment on short arms. Scale bars, 5μm. (TIF) [file pgen.1008968.s004.tif]

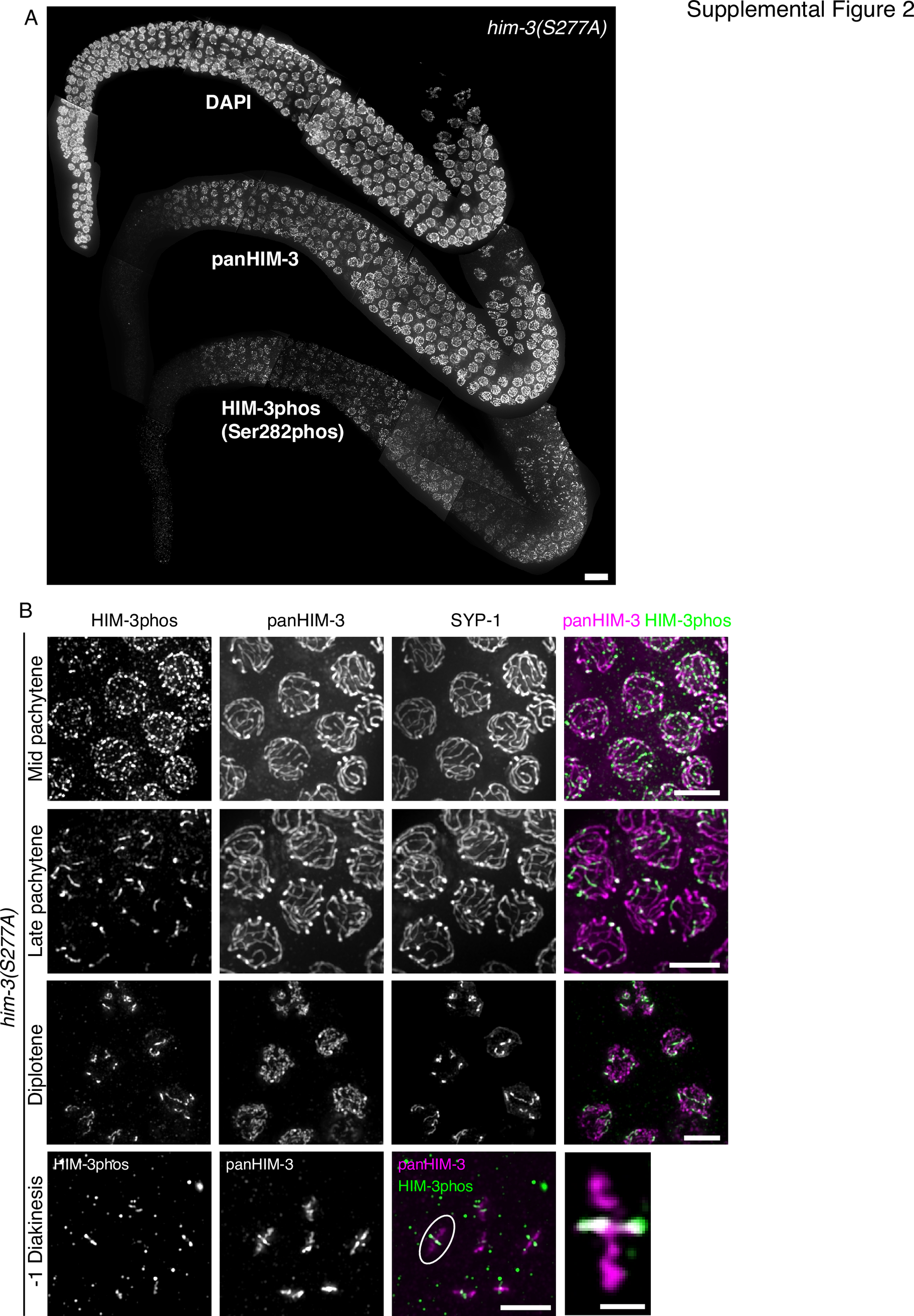

Supplement: S2 Fig — A, Immunostaining of panHIM-3 and HIM-3phos (detecting specifically S282 phosphorylation) antibodies in the him-3(S277A) gonad. Scale bars, 5μm. B. Immunostaining of HIM-3phos (green in merged image), SYP-1, and pan-HIM-3 (magenta in merged image) antibodies in him-3(S277A) mutants. HIM-3phos (Ser282phos) staining is confined to short arms from late pachytene onward. Scale bars, 5μm, and 1μm in the magnified bivalent image. (TIF) [file pgen.1008968.s005.tif]

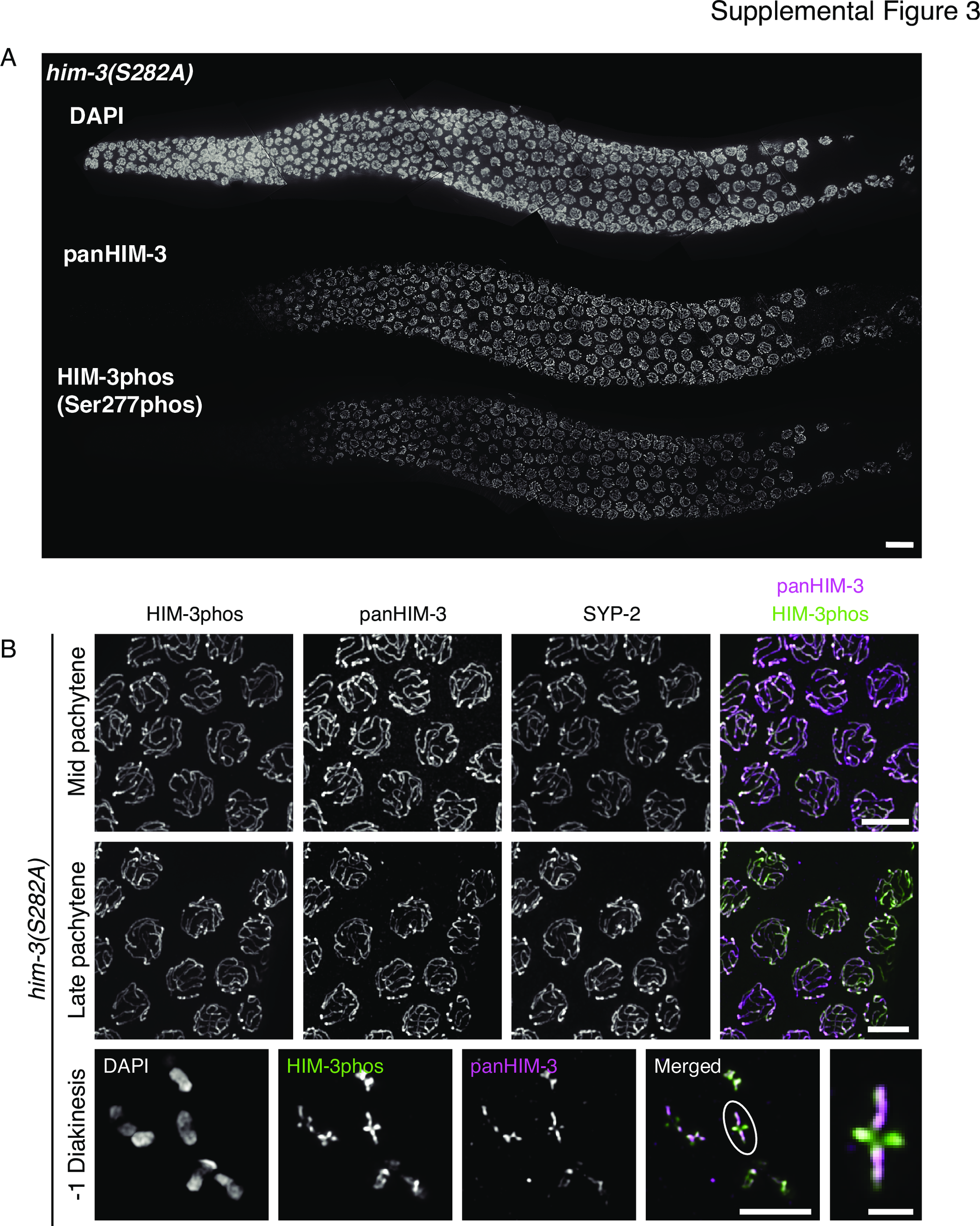

Supplement: S3 Fig — A, Immunostaining of panHIM-3 and HIM-3phos (detecting specifically S277 phosphorylation) antibodies in the him-3(S282A) htp-1::flag gonad. Scale bars, 5μm. B. Immunostaining of HIM-3phos (green in merged image), SYP-2, and pan-HIM-3 (magenta in merged image) antibodies in him-3(S282A) mutants. HIM-3phos (S277phos) staining remains on both short and long arms until -1 diakinesis and does not become enriched on short arms in him-3(S282A) htp-1::flag mutants. Scale bars, 5μm, and 1μm in the magnified bivalent image. (TIF) [file pgen.1008968.s006.tif]

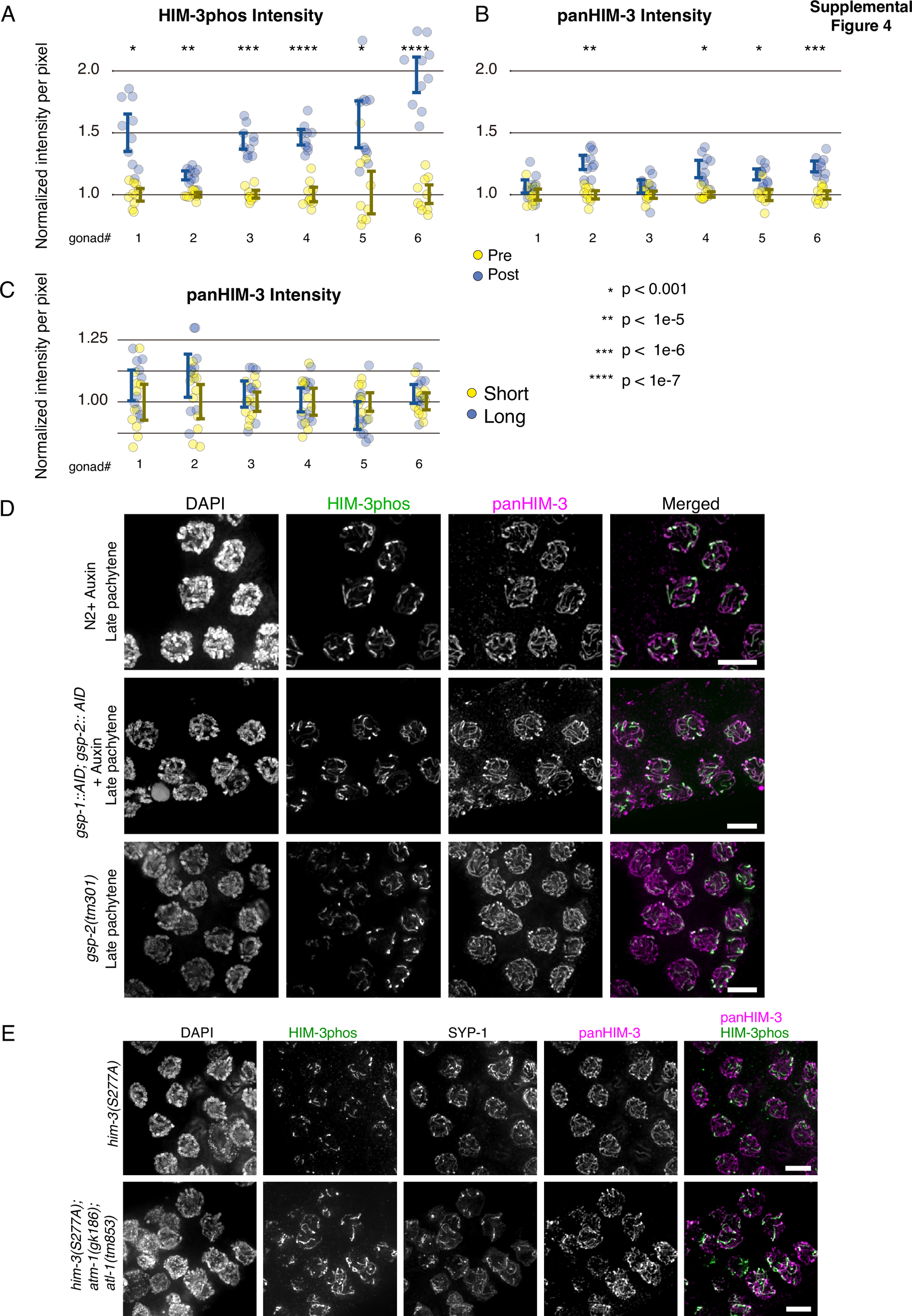

Supplement: S4 Fig — A, B, C: Individual points plotted for each nucleus within each of the 6 gonads scored for HIM-3 intensity quantitation in Fig 3. Each point represents the mean normalized intensity value for 50 pixels picked in one nucleus for the given class. A,B: Comparison of the levels of phosphorylated HIM-3 (A) or panHIM-3 (B) on short arms after partitioning with that of the entire chromosome axis before partitioning within the same gonad. C, Comparison of the levels of panHIM-3 on short arms versus long arms in post-partitioned nuclei. Error bars within each group indicate 95% confidence intervals. “Pre” means pre-partitioned nuclei, “post” means post-partitioned nuclei. Statistical significance was tested by two-tailed t-tests between both conditions in each gonad; p value upper bounds are indicated for each comparison; no value indicates p > 0.05. D, GSP-1/2 are not essential for asymmetric distribution of phosphorylated HIM-3. Immunostaining against phospho-HIM-3 (green in merged image) and pan-HIM-3 (magenta in merged image) in the control (N2 + auxin) and GSP-1/2-depleted gonad (gsp-1(fq51 [gsp-1::degron]); gsp-2 (fq49 [gsp-2::degron]); ieSi38 [Psun-1:: TIR-1::mRuby::sun-1 3'UTR + Cbr-unc-119(+)] + Auxin) as well as in a gsp-2(tm301) null mutant. HIM-3phos staining still disappears from long arms and becomes enriched on short arms in the absence of GSP-1/2. E, Immunostaining of HIM-3phos (green in the merged image), SYP-1 and panHIM-3(magenta in the merged image) in him-3(S277A) (top) and him-3(S277A); atm-1(gk186); atl-1(tm853) (bottom) oocyte precursor cells at late pachytene. Although many polyploid nuclei were seen in the atm-1(gk186); atl-1(tm853) background, SC structures were partially formed, and HIM-3 within these structures was still phosphorylated. Scale bars, 5μm. (TIF) [file pgen.1008968.s007.tif]

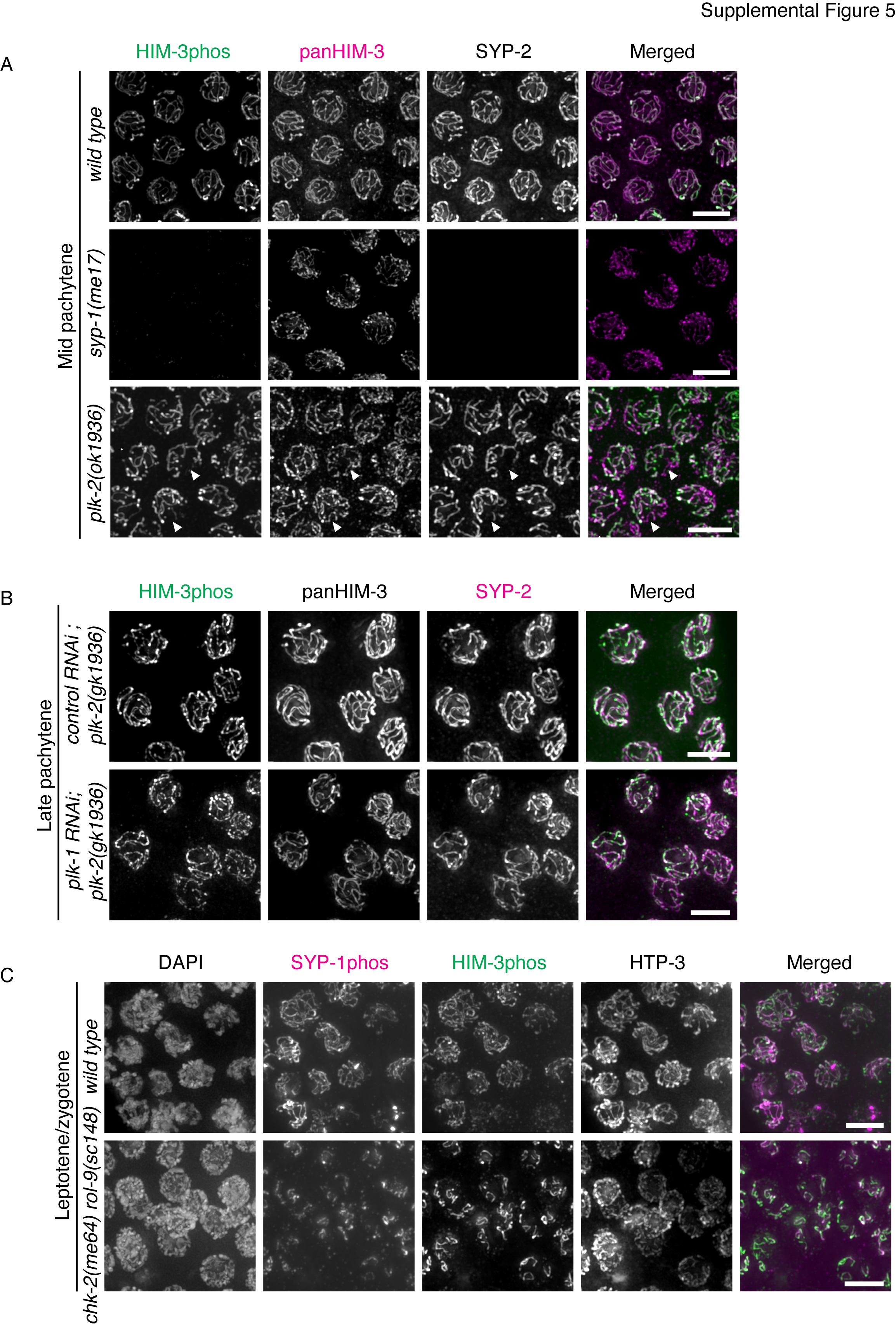

Supplement: S5 Fig — A, Immunostaining of phospho-HIM-3 (green in merged image), pan-HIM-3 (magenta in merged image) and SYP-2 in the indicated genotypes. In the plk-2(ok1936) mutant background (bottom row), arrowheads point to chromosomes that lack synapsis (by α-SYP-2 immunostaining); these same chromosomes also lack HIM-3phos but not pan-HIM-3. B, Immunostaining of phospho-HIM-3 (green in merged image), pan-HIM-3 and SYP-2 (magenta in merged image) in control RNAi (L4440); plk-2 (ok1936) or plk-1 (RNAi); plk-2(ok1936) mutants. HIM-3phos staining is detected on synapsed chromosomes in plk-1 (RNAi); plk-2(ok1936) mutants. C. Immunostaining of HIM-3phos (green in merged image), SYP-1phos (magenta in merged image) and HTP-3 in the chk-2(me64) rol-9(sc148) mutant and N2 wild type. To identify chk-2(me64) rol-9(sc148) homozygous animals, 72 hours post L4 stage adult worms were dissected after verifying the presence of dead eggs due to chk-2 (me64) homozygosity. The rol-9 (sc148) is used to mark chk-2(me64) in cis, and it not expected to cause any meiotic phenotype. HIM-3phos staining is detected on synapsed chromosomes in chk-2(me64) rol-9(sc148) mutants. Scale bars, 5μm. (TIF) [file pgen.1008968.s008.tif]

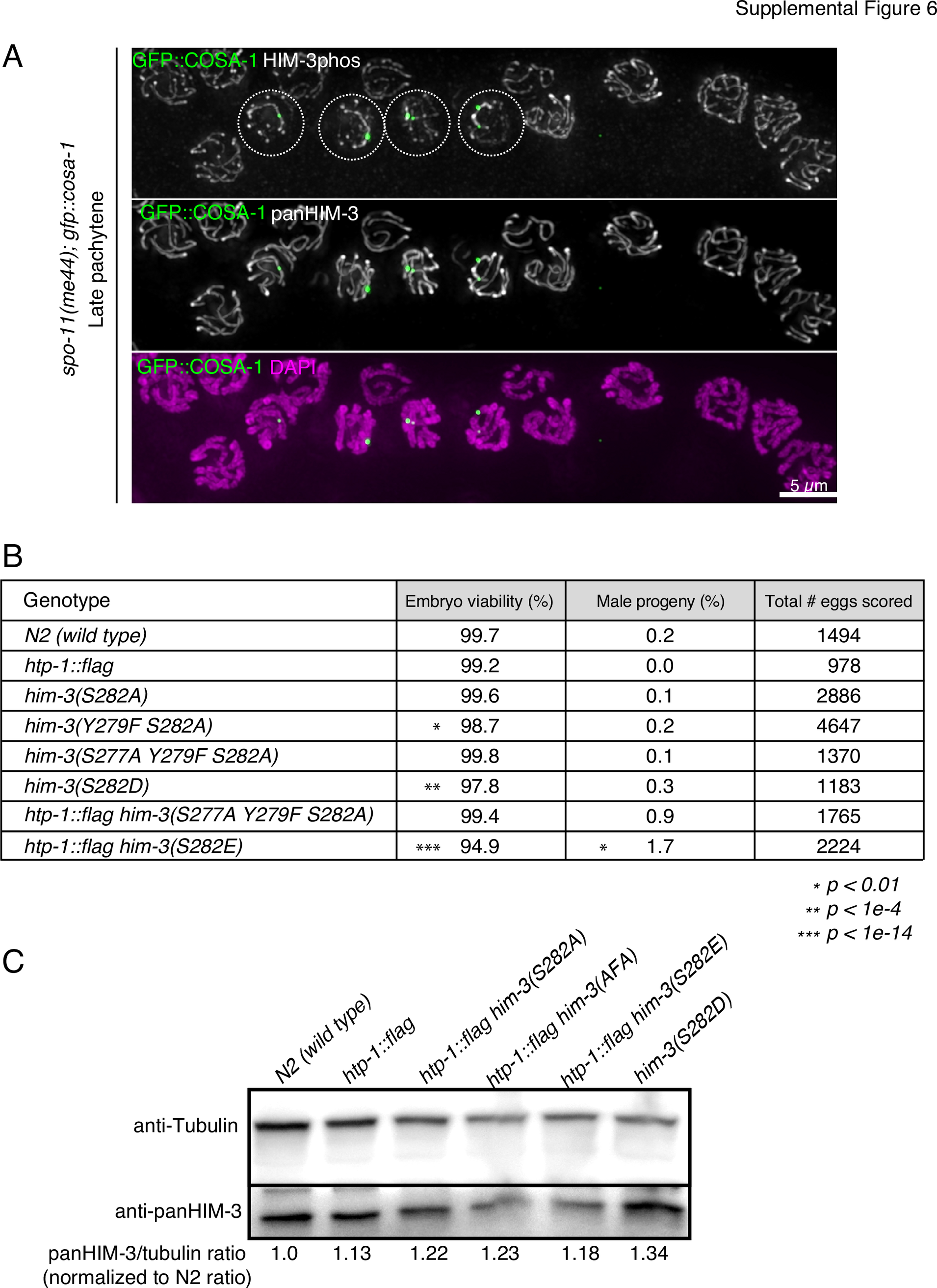

Supplement: S6 Fig — A, Immunostaining of GFP (green in merged image), HIM-3phos and panHIM-3 in late pachytene in spo-11(me44); gfp::cosa-1 mutants. Meiocytes with GFP::COSA-1 are circled with dotted lines and show enrichment of HIM-3phos signals on the chromosomes with GFP::COSA-1. B, Embryonic viability, male progeny production indicating the rate of X chromosome nondisjunction, and total number of scored embryos is shown for the indicated genotypes. p values indicated are from chi-squared test for independence of the embryonic inviability or male counts compared to N2, with Bonferroni correction applied. C, Western blot of control (anti-tubulin) and pan-HIM-3 for adult worms with indicated genotype. Similar levels of HIM-3 proteins are detected in him-3 phospho-mutants compared to control animals. Scale bars, 5μm. (TIF) [file pgen.1008968.s009.tif]

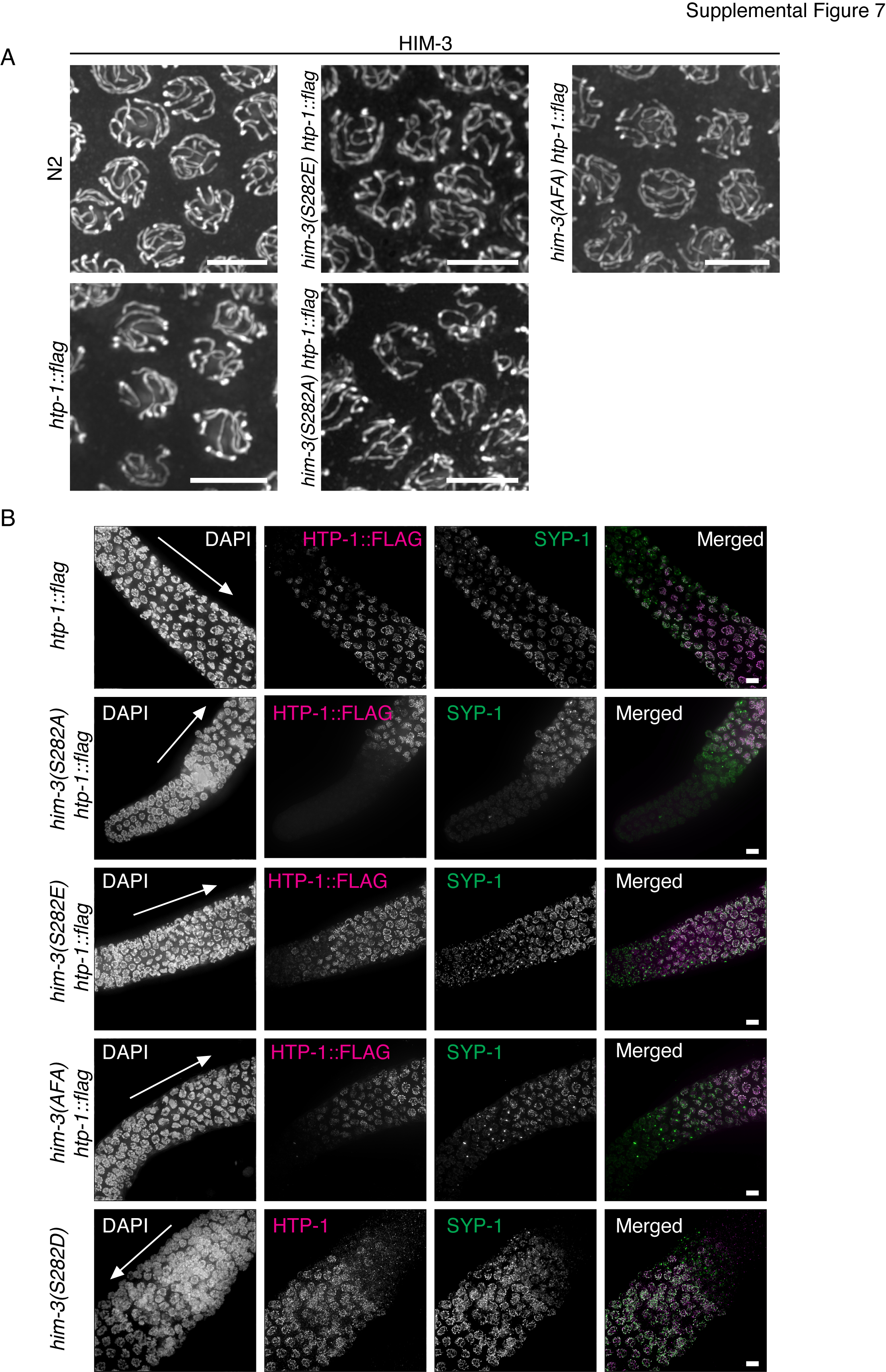

Supplement: S7 Fig — A, Immunostaining of panHIM-3 in pachytene nuclei in the genotypes indicated. The levels and localization of pan-HIM-3 staining in him-3 phospho-mutants appears comparable to that of control gonads. B, Immunostaining with anti-FLAG epitope or HTP-1 (magenta in the merged image) and SYP-1(green in the merged image) in the leptotene/zygotene transition zone in the gonads with indicated genotype. Normal loading of HTP-1 and SYP-1 is observed in him-3 phospho-mutants. Arrows show the direction of meiotic progression in each gonad. Scale bars, 5μm. (TIF) [file pgen.1008968.s010.tif]

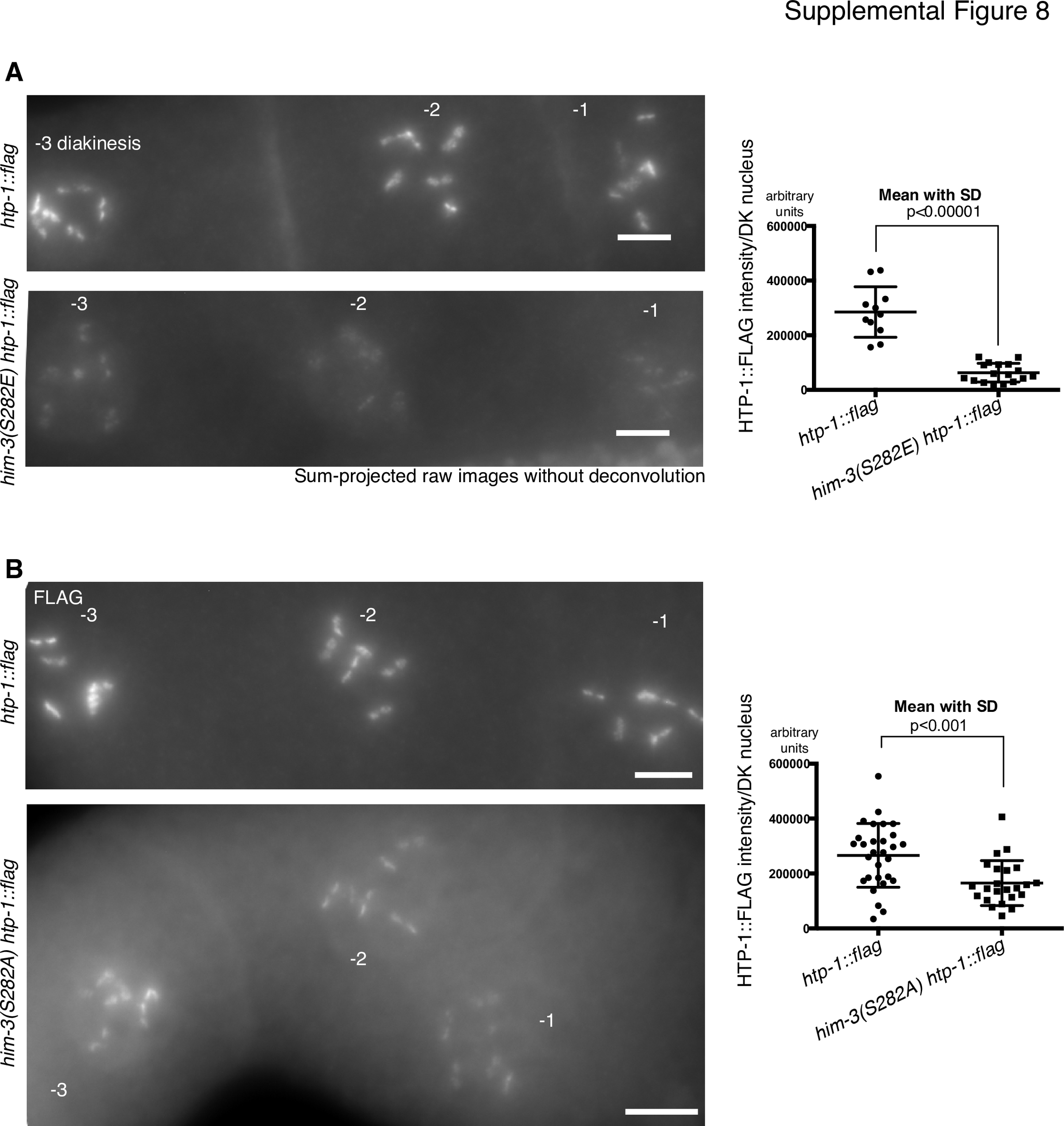

Supplement: S8 Fig — A and B, Immunostaining with anti-FLAG epitope on germlines expressing a transgenic HTP-1::FLAG fusion protein in an otherwise wild-type background or a him-3(S282E) mutant background at diakinesis (A), or in an wild-type background or a him-3(S282A) mutant background (B) stained on the same slide, acquired under the same imaging conditions, and displayed with the same scaling. Images shown are summed projections of raw (not deconvolved) image data. Scale bars, 5μm. Quantitation of the intensity of FLAG::HTP-1 immunostaining in -3 through -1 diakinesis nuclei between the two genotypes in A or B are shown in the right panel. (Significance tested with unpaired two-tailed t-test after passing Shapiro-Wilk normality test, p<0.0001 for A and p<0.001 for B) Scale bars, 5μm. (TIF) [file pgen.1008968.s011.tif]

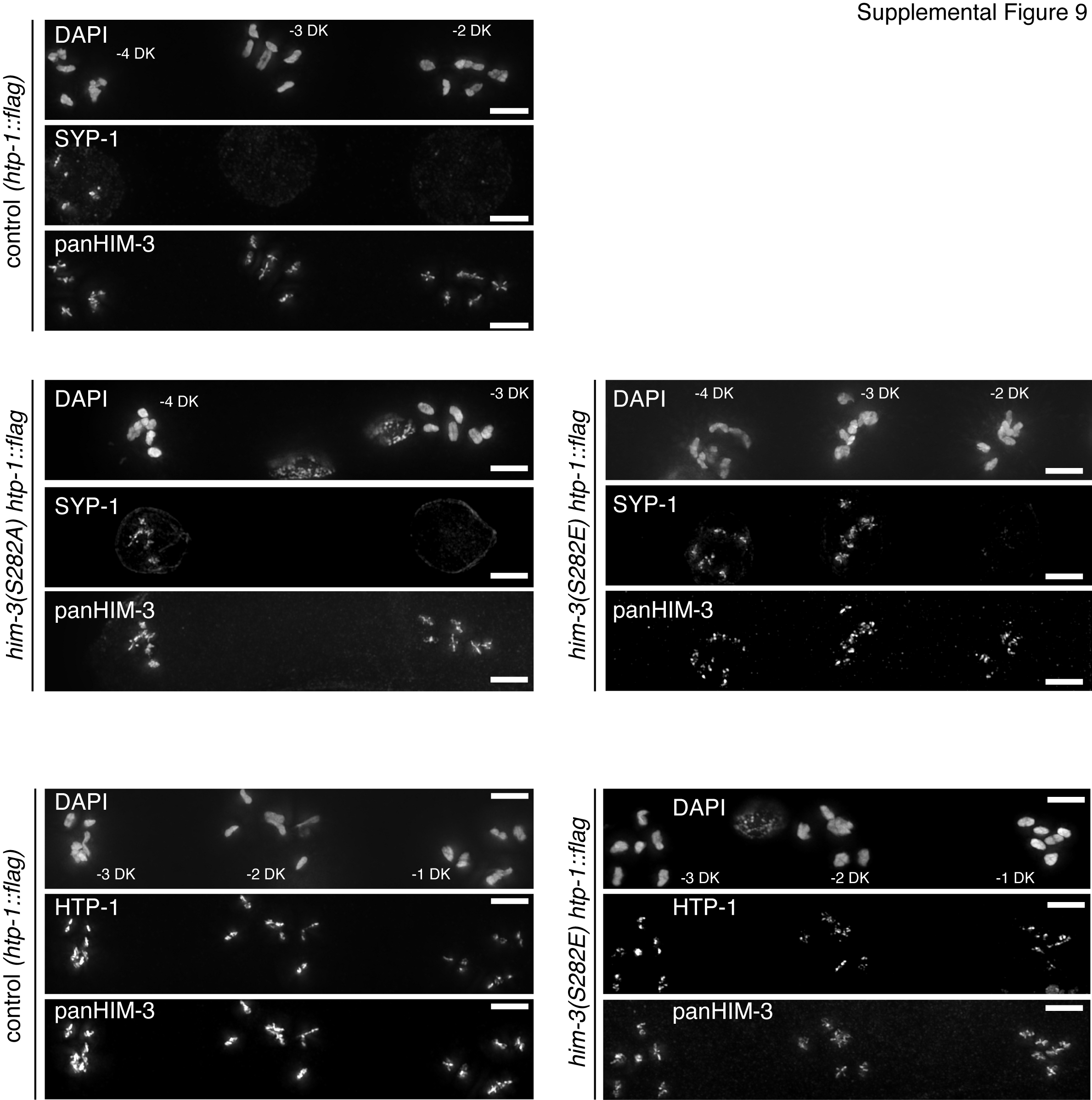

Supplement: S9 Fig — Immunostaining of panHIM-3 and either SYP-1 or FLAG in control (htp-1::flag), him-3(S282A) htp-1::flag and him-3(S282E) htp-1::flag mutant gonads. The same SYP-1 panels of him-3(S282A) htp-1::flag and him-3(S282E) htp-1::flag mutants as well as the HTP-1 panel of him-3(S282E) htp-1::flag mutant are presented in the main Fig 6. Scale bars, 5μm. (TIF) [file pgen.1008968.s012.tif]

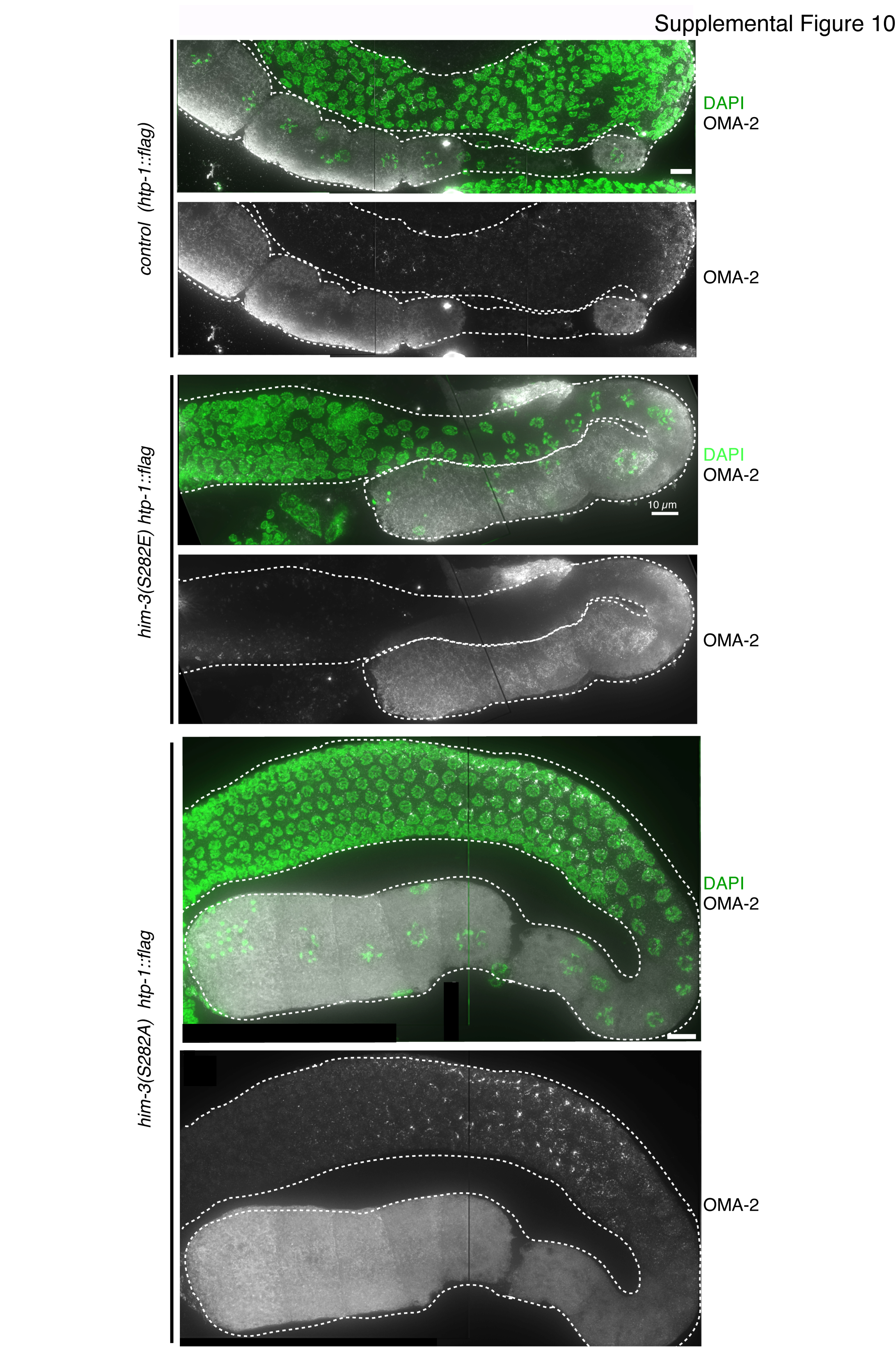

Supplement: S10 Fig — Immunostaining of OMA-2 in htp-1::flag control, him-3(S282E) htp-1::flag and him-3(S282A) htp-1::flag mutant gonads. DAPI is shown in green in merged images. Scale bars, 5μm. (TIF) [file pgen.1008968.s013.tif]

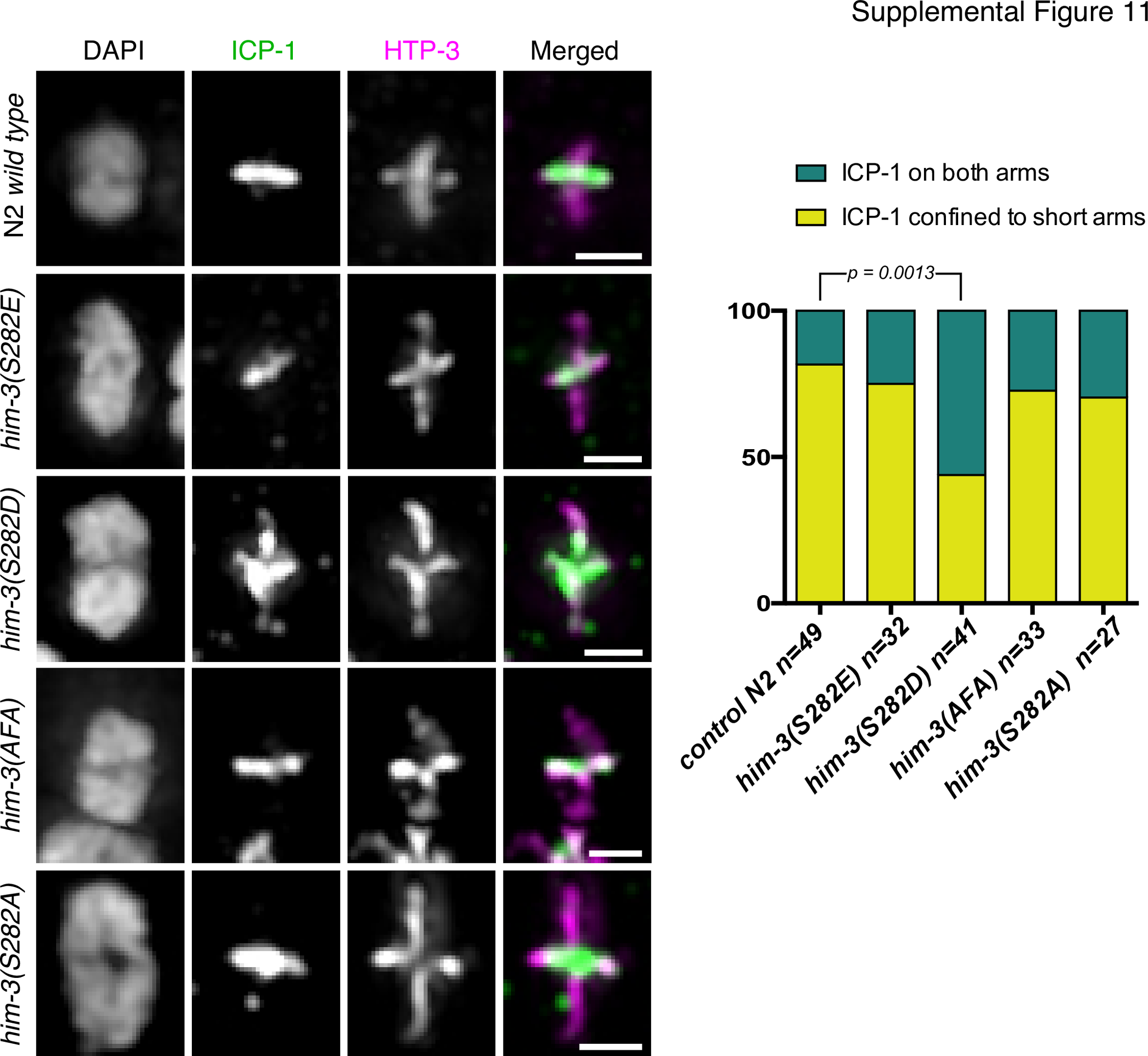

Supplement: S11 Fig — Shown are partial maximum-intensity projections of single chromosomes oriented so the long arms are vertical. Scale bars, 1μm. Quantification of ICP-1 localization classes (confined or unconfined) is shown at right, along with numbers of nuclei scored. Data shown are from at least 2 independent biological replicates. p-value of Fisher’s exact test for independence after correction for multiple comparisons is shown. (TIF) [file pgen.1008968.s014.tif]

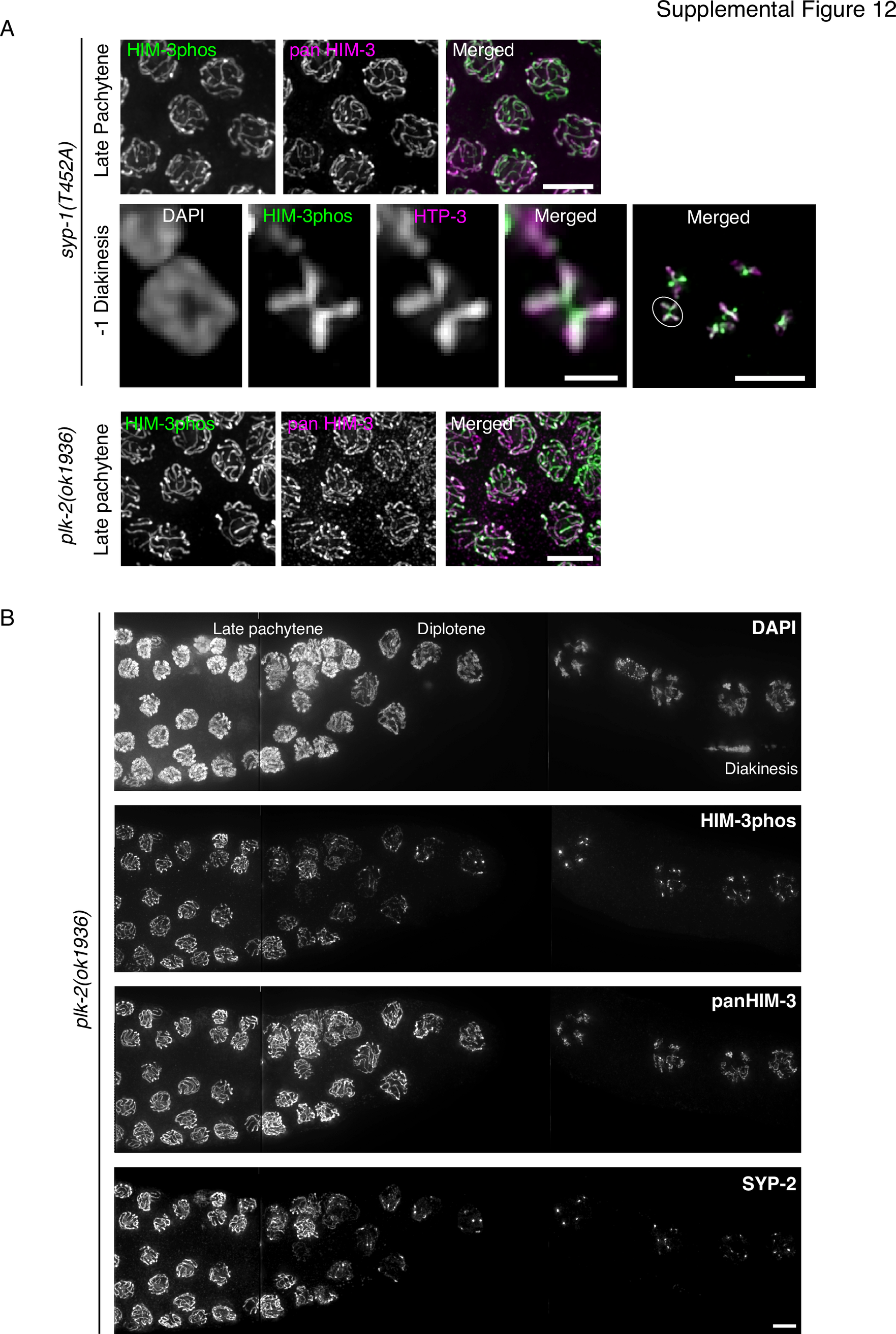

Supplement: S12 Fig — A, Asymmetric enrichment of phosphorylated HIM-3 is abrogated in syp-1(T452A) or plk-2(ok1936) mutants. Immunostaining of phospho-HIM-3 (green in merged images) and pan-HIM-3 (magenta in merged images) in the indicated genotypes showing phospho-HIM-3 failing to partition the short arm. Partial Z projections are shown for all images. B, Phosphorylated HIM-3 colocalizes with SYP-2 along the entire SC in late pachytene, and to presumptive CO designation sites in diplotene in plk-2 (ok1936) mutant gonads. Scale bars, 5μm, and 1μm for the magnified bivalent images. (TIF) [file pgen.1008968.s015.tif]

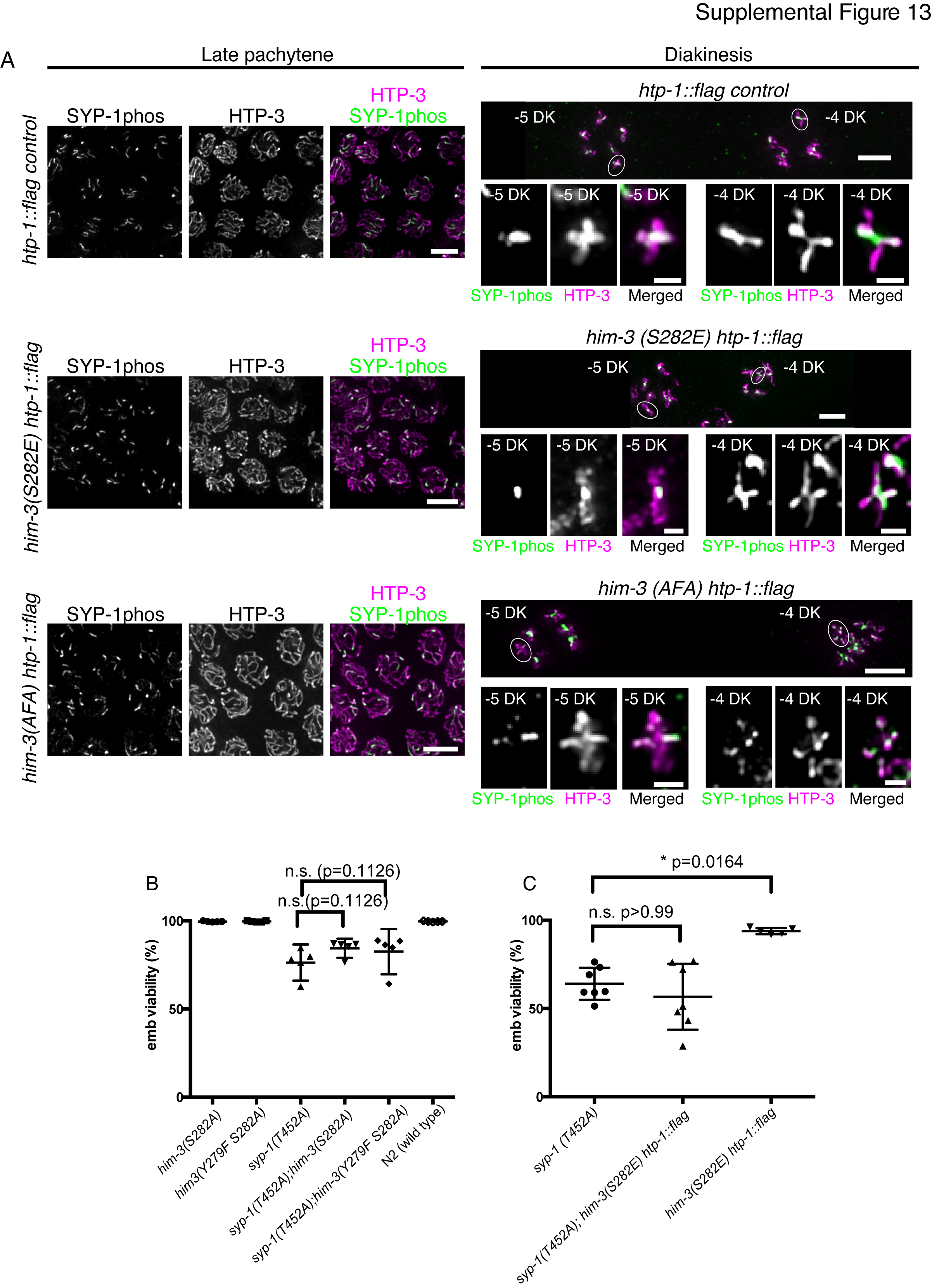

Supplement: S13 Fig — A, Immunostaining of phospho-SYP-1 (green in merged images) and HTP-3 (magenta in merged images) in the indicated genotypes. For both A and B, individual diakinesis chromosomes displayed are circled in the overview images. In him-3(S282E) or (AFA) mutants, although SYP-1phos staining is confined normally to short arms from late pachytene up to -5 diakinesis, SYP-1phos signals reappearing on long arms are occasionally found specifically in -4 and -3 diakinesis nuclei, presumably due to de novo phosphorylation activity by yet-unidentified kinase in mature oocytes. Scale bars: individual diakinesis chromosomes, 1μm; all others 5μm. B and C, Percentage of embryonic viability among the self-progeny of worms with the indicated genotypes. Total number of eggs scored are: him-3(S282A): 1583, him-3(Y279F S282A): 1613, syp-1(T452A)1129, syp-1(T452A); him-3(S282A): 1449, syp-1(T452A); him-3(Y279F S282A): 1336 N2: 1494 in graph B, and syp-1(T452A): 1413, syp-1(T452A); him-3(S282E) htp-1::flag: 1371, him-3(S282E) htp-1::flag:1218 in graph C. Mean with 95% confidence intervals is indicated in the graph. No statistical significance was detected between the syp-1(T452A) single mutant with syp-1(T452A); him-3 (S282A or Y279F S282A or S282E) double mutants using Kruskal-Wallis analysis with Dunn’s multiple comparison test. (TIF) [file pgen.1008968.s016.tif]

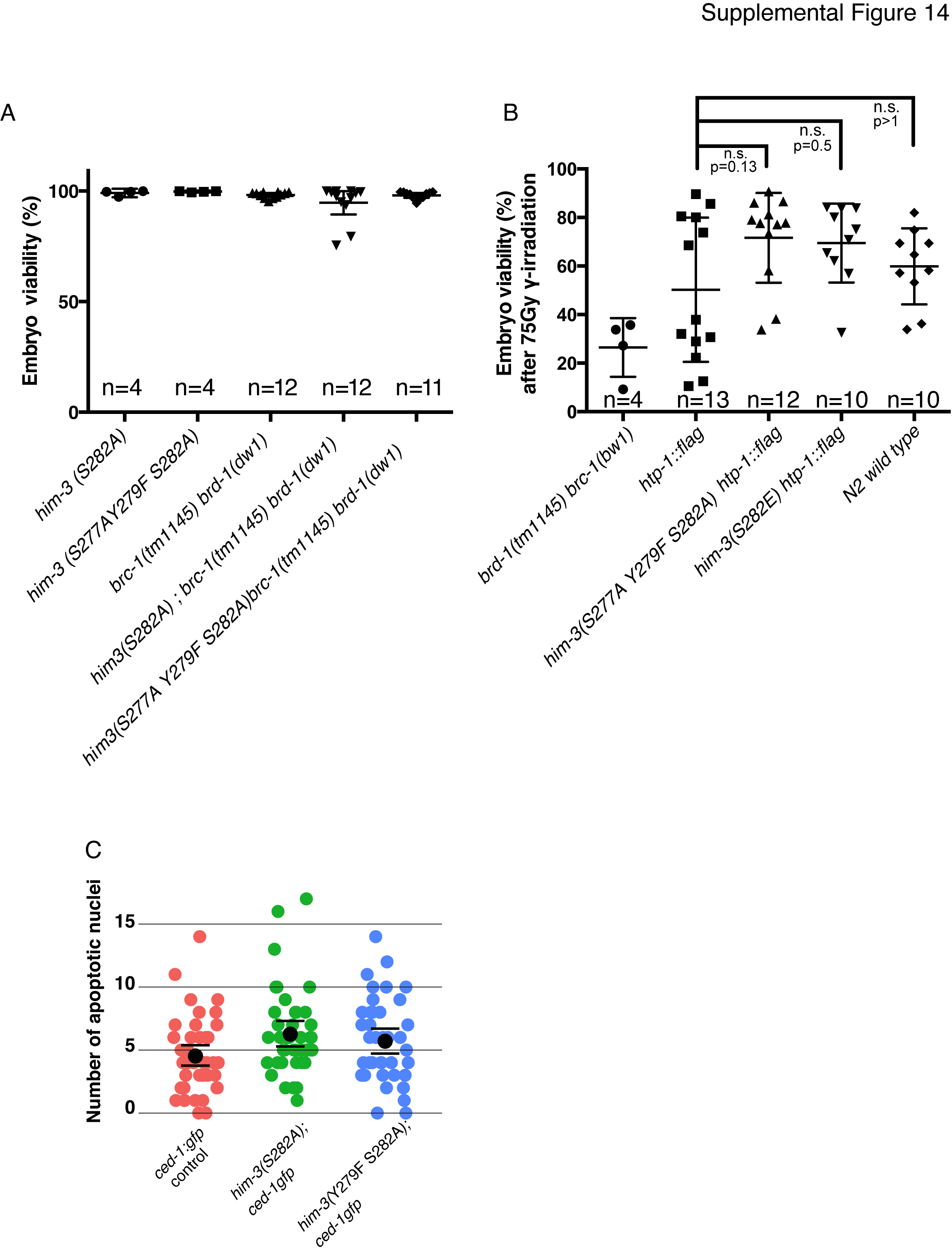

Supplement: S14 Fig — A, Percentage of embryonic viability among the self-progeny of worms with the indicated genotypes. For each column, n indicates the number of maternal worms. Total number of eggs scored are: him-3(S282A):1185, him-3(AFA): 1370, brc-1(tm1145) brd-1(dw1): 3013, him-3(S282A); brc-1(tm1145) brd-1(dw1): 2178, him-3(AFA); brc-1 brd-1: 2443 eggs. Mean with 95% confidence intervals is indicated in the graph. The him-3 non-phosphorylatable mutations did not lower embryonic viability when sister-chromatid-mediated homologous recombination is prevented in the brc-1(tm1145) brd-1(dw1) background. B, Percentage of embryonic viability among the self-progeny of P0 worms after 75 gray γ-irradiation at L4 stage. The brc-1(tm1145) brd-1(dw1) mutant was used as a positive control, which shows reduced embryonic viability upon gamma irradiation. Total number of eggs scored are: brc-1(tm1145) brd-1(dw1): 646, htp-1::flag: 1178, him-3(AFA) htp-1::flag: 1896, him-3(S282E) htp-1::flag: 1233 eggs. Mean with 95% confidence intervals is indicated in the graph. The him-3 phospho-mutations did not lower embryonic viability upon exogenous DNA damage using Kruskal-Wallis test with Dunn’s multiple comparison. C. Scoring of apoptotic nuclei in him-3 phospho-mutant backgrounds. The number of CED-1:GFP-positive nuclei for each condition is plotted with mean and 95% confidence intervals. The him-3 non-phosphorylatable mutations did not increase the number of apoptotic nuclei. (TIF) [file pgen.1008968.s017.tif]

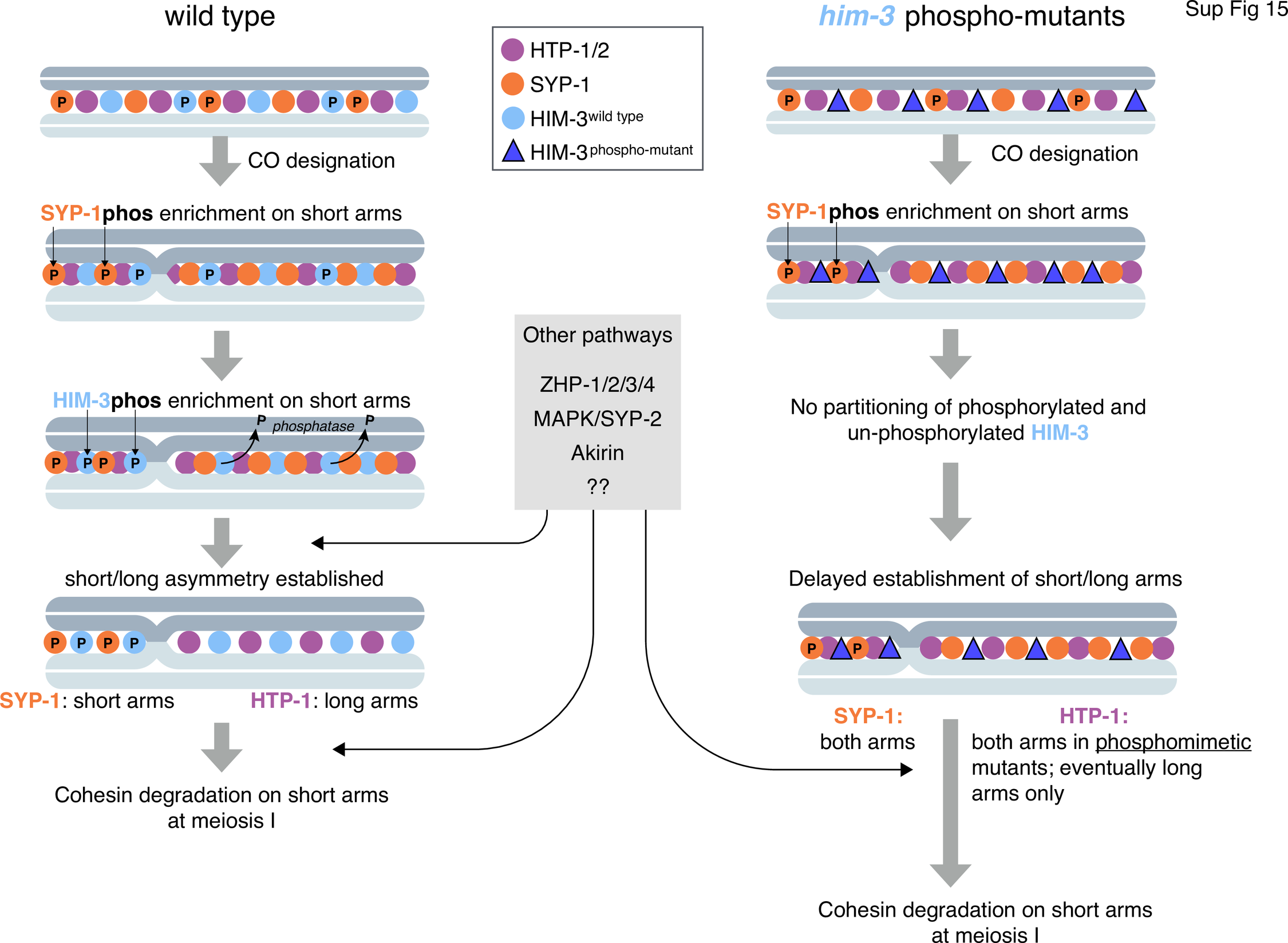

Supplement: S15 Fig — (TIF) [file pgen.1008968.s018.tif]
