# Supplementary material for: Phosphoregulation of HORMA domain protein HIM-3 promotes asymmetric synaptonemal complex disassembly in meiotic prophase in Caenorhabditis elegans
Source: PLoS Genet. 2020 Nov 11;16(11):e1008968. doi: 10.1371/journal.pgen.1008968 (PMC7717579; doi:10.1371/journal.pgen.1008968)
Supplement: Attachment — Submitted filename: HIM-3_Phosphorylation_Manuscript_point-response.docx [file pgen.1008968.s019.docx]

**Responses to comments from the Editor:**

**(our answers in blue text):**

*Comments from the Editor*

Should you decide to revise the manuscript this should include, but not be restricted to, the following: 1) completing the analysis of known C. elegans meiotic kinases- such as by testing HIM-3 phosphorylation in plk-2; plk-1 double mutants, 2) testing the model of redundancy of establishment of short and long arms by the analysis of the appropriate double mutant, 3) The reviewers also point to some cases in which data is not shown but described, validation of reagents that is missing, and insufficient experimental detail description- please provide all the data stated but not shown in the supporting material and the missing experimental details and reagent validation in the methods section,

We agree that the identification of one or more kinases phosphorylating HIM-3 would be an extension to our manuscript that would make it more impactful. While it is not feasible to test all known meiotic kinases for the current manuscript, we have appended an analysis of those we hypothesized to have the greatest chance of phosphorylating HIM-3: *plk-1(RNAi); plk-2* double mutants and *chk-2* mutants. We now show HIM-3phos staining in these backgrounds in Supplemental Figure 5.

We have added an analysis of double mutants of *syp-1 (T452A)* non-phosphorylatable and *him-3* non-phosphorylatable/phosphomimetic mutations and have shown that the *syp-1(T452A)* mutation is epistatic to our *him-3* phospho-mutants. This is consistent with our cytological observation that SYP-1 phosphorylation is required for partitioning of phos-HIM-3 to short arms. As we detail in the responses to reviewers, we also attempted to experimentally address the redundancy of short and long arm establishment in *htp-3;him-3* double closure motif mutants, but difficulties with strain construction precluded the completion of this experiment.

4) all data quantified should have the appropriate statistics clearly stated: significant p values indicated on figures, and a section in materials and methods that will describe the statistical analysis (e.g., which normality test was used before t-test was performed?) for all quantified data set, including stacked bar graph data and data in tables.

We have newly indicated all relevant p values in either the figures themselves, or figure legends, for Fig. 1C, Fig. 6C, Sup.Fig. 4ABC, Sup.Fig 6B, Sup. Fig. 8, Sup.Fig.11, Sup.Fig. 13BC, and added description of the statistical analysis in the corresponding figure legends. All the numerical values for the graphs are provided in Sup.Table 3.

We now describe all methods referred to in the paper, as detailed in the point-by-point responses.

Further responses to the Editor’s comments are detailed point-by-point below.

In addition I have the following critiques as editor that I would like you to respond to:

• The image emerging from the analysis of the HIM-3 mutants is complex. Adding a Sup figure explaining the author’s model and the mutants’ phenotypes will be very helpful for the reader.

We have added a figure (Supplemental Figure 15) to describe the phenotypes.

• S4A- Why does the htp-1 flag tag in him-3(S282E) background show emb, but not on its own?

Also, in S7b the flag tag shows emb after IR (assume more the WT, but there is no control). These 2 observations imply that the tag is not fully functional.

We think it is the S282E mutation that causes Emb in the *htp-1::flag* background, and not vice versa, since (1) the *htp-1::flag* tag does not increase embryonic inviability by itself or in any other background we have observed, and (2) the other phosphomimetic mutation S282D in a wild-type background also shows Emb, and (3) the delay in asymmetry establishment in S282E is a plausible cause of (mild) embryonic inviability. Further, we have repeated the irradiation experiment with N2 wild type and have shown that there is no significant difference in post-irradiation embryonic inviability between *htp-1::flag* and N2 (previously S7B, now S14B in the revised manuscript).

• S7A and B- Wild type control is missing.

We have added an N2 control to Sup Fig 14 (previously Sup.Fig. 7) B by repeating the experiment. For the experiment in A, the purpose is to test whether the *him-3* mutants are synthetic Emb in the *brc-1; brd-1* background; as the single *him-3* phosphomutants showed their usual high (near-WT) viability, we have omitted an N2 control here as superfluous.

• S7A -Why does the htp-1::flag (on its own) show emb after IR and why is it rescued by the mutants?

Previously we failed to indicate p values in the S7B (current S14B), and this gave a wrong impression that *him-3* mutations rescued embryonic viability. However, while there is variability in Emb phenotypes after 75Gy γ-irradiation, there is no significant difference between *htp-1::flag* on its own and in combination with any of the tested phosphomutants of *him-3*. Now p values and details of statistical analysis are provided in the S14B graph and its figure legend.

• S4- Shows just S282E, there is no data showing the effect on HTP localizations level in other mutants.

We have now quantitatively examined HTP-1::FLAG levels in S282A as well and included in the supplemental figure 8. We also provided images of HTP-1::FLAG (anti-FLAG) or HTP-1(anti-HTP-1) staining in control, *him-3(S282A), him-3(S282E), him-3(AFA)* and *him-3(S282D)* mutants to show that the timing of HTP-1 loading is normal in the leptotene/zygotene transition zone in all the *him-3* mutants in supplemental figure 7B.

• Images of FAF mutants 6B shows axis/bivalents partially detaching- is this a common phenotype?

Diakinesis chromosomes at high resolution show a range of axis protein localizations including continuous, separate, and “hyperbolic” (HTP-3 staining in AFA mutant in 6B) depending on the orientation of the chromosome relative to the optical axis; we have re-examined bivalent morphology in *him-3(AFA*) and control, and we do not think that this morphology (AFA) is a departure from normal observations.

• Based on the text of figure 6, the mislocalization of HTP-1 and SYP-1 in the HIM-3 mutants is eventually resolved. The authors need to present the data supporting this statement in figure 6 (not sup).

We have added images of the eventual confinement of HTP-1 to long arms, and the eventual disappearance of SYP-1 in *him-3* mutants in Fig 6D and supplemental figure 9 (Sup.Fig. 9 provides controls and more detailed information of Fig6D).

• Typos:

o 1um labeling in 6B is misplaced.

o S7b is flag::htp-1 supposed to be htp-1::flag?

o Fig 4B, 5B scale bar moved up in the bottom panels.

o S3- italics where it should not be needed on labels.

These have all now been fixed. We do appreciate your attention in pointing out these errors.

**Responses to Reviewer's Comments**

(our responses are in blue text; line numbers refer to the clean document).

***Comments from reviewers:***

***Reviewer #1:*** This manuscript “Phosphoregulation of HORMA domain protein HIM-3 promotes asymmetric synaptonemal complex disassembly in meiotic prophase in C. elegans” presents insights into the role of phosphorylation in regulating meiotic chromosome remodeling and SC disassembly. Meiotic HORMA domain proteins in C. elegans, HIM-3, HTP-1, -2, and -3, form hierarchical assemblies through binding of their HORMA domains to cognate peptide motifs termed “closure motifs” and play essential roles during meiosis. Here, the authors identified that HIM-3 is phosphorylated at its closure motif, thereby disrupting its binding to HTP-1/2 in vitro. Using phospho-specific antibody, the authors showed that HIM-3 phosphorylation occurs along synapsed axes in early meiotic prophase, but becomes enriched on the short arm upon crossover formation. It was further demonstrated that HTP-1 release and SC disassembly are delayed in worms expressing phospho-mimetic mutations of HIM-3, supporting the role of HIM-3 phosphorylation in axis remodeling.

While it is clear that the phosphorylation at S282 dramatically reduces HTP-2 binding in vitro, the results from in vivo analyses using him-3 mutants are somewhat confusing. For example, both him-3(S282E) and him-3(S282D) worms contain phospho-mimetic mutations and display similar defects in SC disassembly (Figure 6C); however, they show distinct phenotypes in ICP-1 distribution (Figure S5). Can the authors explain why this is the case?

We agree that the ICP-1 distribution disparity between S282E and S282D is puzzling. We have re-examined the ICP-1 data, and still come to the same conclusion. We speculate that S282D may be a slightly better phosphomimic than S282E, which in this case leads to a stronger phenotype. If this is the case, it would imply that CPC localization is sensitive to HIM-3 phosphorylation and is something we will explore in future work.

Another confusing part (to this reviewer) is in Discussion to explain how the delay in HTP-1 release is observed in him-3 phospho-mimetic mutants, but not in phosphorylation-defective mutants. The authors speculate that, in him-3 phospho-mimetic mutants, HTP-1/2 are likely recruited to the axis through binding HTP-3 motifs, which may result in less efficient dissociation. However, this same scenario will be applied to axis-recruited HTP-1/2 in non-phosphorylatable mutants, where a pool of HTP-1/2 is bound to HTP-3 motifs and another pool is bound by HIM-3. The readers will greatly benefit from some clarification and potentially further experimentation (e.g. what will happen if him-3 phospho-mutations are combined with HTP-3 mutations where HTP-1/2-binding closure motifs are mutated?).

We have attempted to clarify our admittedly speculative model with explanatory text (lines 620-625). Essentially our speculative model is that “overloading” HTP-1/2 onto HTP-3 (i.e., HTP-1/2’s HTP-3:HIM-3 binding ratio is increased) may make it more difficult to remove. We have also attempted to address the question experimentally. We have obtained the *htp-3(2GK)* mutation (*htp-3(tm3655) I; Si(htp-3(G490K,G728K)::gfp; unc-119(+)]II*)) which mutates the HTP-1/2 closure motifs and have attempted several times to create a double-mutant strain with our HIM-3 phosphomutants (with *htp-1::flag* in the background for cytology). Unexpectedly, when we cross *him-3(S282A) htp-1::flag* mutants to *htp-3(2GK)* mutants, although the F1 heterozygotes are fully viable and fertile, we are unable to recover *htp-3(tm3655) I; Si(htp-3(G490K,G728K)::gfp; unc-119(+)]II; htp-1::flag him-3(S282A) IV* all-homozygous animals among the F2 progeny even when we pick a number of F2 animals overwhelmingly sufficient to obtain homozygous animals assuming Mendelian inheritance. We are puzzled by this apparent embryonic lethality but have had to opt out of further pursuit of this experiment due to the limited time frame.

Other major points:

1. Please describe how the HIM-3 phospho-specific antibody was generated and purified and how the phospho-specificity has been validated in Materials and Methods.

Thanks for pointing out the oversight. This has now been added to the methods section.

2. The phospho-HIM-3 antibody was raised against a phospho-peptide containing phosphates at both S277 and S282. Thus, it is unclear whether the signal reflects the phosphorylation event at both residues or whether it recognizes either pS277 or pS282. This has to be established as an important validation of the antibody specificity. Have the authors tested the phospho-HIM-3 antibody in him-3(S282A) mutants? Typically, a phospho-epitope is very tight around the phosphorylated residue (if purified correctly). It will help interpret the temporal and spatial pattern of HIM-3 phosphorylation at S282, the residue that the authors mostly focused on in this paper.

We thank the reviewer for this suggestion. We have performed staining with this antibody in both S277A and S282A single mutants to define the specificity, and now present the results in Figures S2 and S3. In short, the polyclonal antiserum recognizes both residues. We found that in *him-3(S277A)* mutants, short and long arm asymmetry is established normally, and S282phos becomes confined more rapidly and completely to short arms than the combined S277/S282phos signal in wild type. Because of the slight difference between “S282phos only staining” (in S277A mutants) vs. “S277phos/S282phos combined staining” (in wild type), we think that S277phos is the signal faintly persisting on long arms until -2 diakinesis in wild type.

In contrast to S277A mutants, the S282A mutation delays short/long arm establishment (as shown in Fig. 6), and we found that S277phos staining remains along the entire length of the chromosome (i.e., never partitions). This shows that phosphorylation status at S282 not only affects SYP-1 or HTP-1 asymmetry but affects S277 dephosphorylation on its own. These data revealed a potential dependence in the dynamics of these two phosphosites which will be interesting to explore in future work.

3. What is the binding affinity of the peptide harboring phospho-mimetic mutations (S282E or S282D) in vitro? It will be important to test whether these mutations truly mimic phosphorylation. It will also help interpret the phenotype seen in worms expressing HIM-3(S282E vs. S282D).

Unfortunately it is no longer feasible for us to perform further FP assays, since we don’t have any more HTP-2 protein (prepared by the Corbett lab team). Scott Rosenberg who purified the initial batch of HTP-2 has left their lab; they have attempted to repurify HTP-2 proteins multiple times (a complicated procedure requiring co-expression of multiple proteins) since then but failed to do so.

Minor points:

1. The authors showed that HIM-3 phosphorylation depends on the SC formation, but is still present along synapsed axes in plk-2 mutants. How about plk-2; plk-1 double mutants? In C. elegans, PLK-1 is known to substitute for PLK-2 function in the germline.

We have now examined HIM-3phos staining in *plk-1(RNAi); plk-2* double mutants and show this data in Sup. Fig5; in short, the observations do not differ from *plk-2* single mutants.

2. The authors show that partitioning HIM-3 phosphorylation on the short arm depends on SYP-1 T452 phosphorylation. What does the phospho-HIM-3 staining look like after crossover formation in plk-2 mutants? Does it still exhibit short arm enrichment?

We now show phospho-HIM-3 in *plk-2* mutants throughout late prophase in **Supplemental Figure 12**; as has been previously demonstrated for the SYP proteins in *plk-2*, phospho-HIM-3 localizes to the entire chromosome length (i.e., does not partition) until diplotene, at which point it disappears from most of the chromosome, remaining only at a single point (the putative crossover). Phospho-HIM-3 never shows short arm enrichment in *plk-2* mutants.

3. What would be the significance of HIM-3 phosphorylation along synapsed axes in early meiotic prophase?

We have attempted to find a role for early phospho-HIM-3 by examining the synapsis checkpoint and synthetic lethality with *brc-1*, but have not determined a conclusive role for the early HIM-3 phosphorylation. One possibility we mention in the discussion section (lines 625-630) is that a fraction of HIM-3 protein may need to be phosphorylated and thus remain unoccupied by HTP-1/2 for timely polymerization of SYP-1. This is because a previous study has shown that disrupting the two HTP-1/2 binding sites of HTP-3 causes HTP-1/2 to bind exclusively to HIM-3 and leads to a delay in synapsis [(Kim et al. 2014).](https://paperpile.com/c/y4DJc6/BfZW)

***Reviewer #2***:

The manuscript by Sato-Carlton et al., describes the identification and functional characterization of HORMA axis protein, HIM-3 phosphorylation, in C. elegans meiosis. Using a combination of biochemistry, cell biology and genetics, the authors find that 1) HIM-3 is phosphorylated on the conserved C-terminal closure motif, 2) phosphorylation blocks HIM-3 binding to HTP-2 in vitro, leading to a model whereby phosphorylation contributes to removal of HTP-1/2 from the short arm, 3) phosphorylated HIM-3 becomes enriched on the short-arm of the bivalent dependent on the formation and number of crossovers, 4) phosphorylation of HIM-3 plays a role in the timely disassembly of the SC. This is a well-executed study that provides insight into the role and regulation of phosphorylation on SC axis and central region components and illustrates the complex and redundant nature of establishing chromosome domains for accurate chromosome segregation. Please consider the following:

1. The introduction would benefit from some rewriting. Here are a couple of examples/suggestions: page 2: In contrast, organisms with holocentric . . . must define two separate domains . .” (vs. separation) and “At meiosis I, cohesion . . . on long arms persists until degraded . . .(remove is finally). Page 3: The way the ZHP section is written implies that ZHP-1 is a partner with ZHP-3 and ZHP-2 is a partner with ZHP-4; this is incorrect.

Thanks for pointing out where the writing was unclear; we have rewritten these sections as indicated.

2. Figure 2: please indicate in the legend that the cruciform structure observed is dependent on orientation. In 2C, it appears that there is weaker staining of phosphoSYP-1 immediately adjacent to the COSA-1 foci compared to phosphoHIM-3 – is this consistent? Any thoughts?

We have added the explanation about the cruciform structure in Figure 2’s legend.

The image showing SYP-1phos signals seemingly enriched from the telomere side rather than the CO is within the range of variability that we see, but it does not seem to be a consistent feature of SYP-1phos or a consistent difference between HIM-3phos and SYP-1phos.

3. Figure 5: I would like to see a discussion on how the finding that the total number of COs promotes asymmetric distribution of phosphoHIM-3 to the short arm relates to the surveillance pathway that individually monitors crossovers on each chromosome and maintains checkpoint signaling until all chromosomes have a CO as described by Yanowitz and colleagues (Machovina et al., 2016). Did the authors consider treating spo-11 mutants with IR and examining CO numbers and phosphoHIM-3 enrichment to the short arm?

This is a good point that we did not give enough space to; we have added discussion on this point to the Results (lines 360-365) and the Discussion (~line 637). We did consider the *spo-11*/IR experiment but decided to use *dsb-2* instead, on the rationale that γ-ray-induced lesions, while capable of rescuing bivalent formation, are (often) not the same kind of break as is made by SPO-11; moreover, the range of DSB numbers created (by SPO-11) in the *dsb-2* background is ideal for analyzing all the conditions needed.

4. Figure 6: Phenotypic consequence of HIM-3 phosphorylation. Does the delay in SYP and HTP-1/2 removal from chromosome domains affect the timing of cohesin removal? Given the subtle phenotypes, analysis of the double syp-1-nonphospho; him-3-nonphospho mutant would be informative. If syp-1-nonphospho mutant is epistatic, it will support the model. Alternatively, if there is any synergism, this will also be a meaningful result. It would also be interesting to combine the him-3 nonphospho mutant with the syp-2 nonphosho mutant as well as combining him-3 nonphospho mutant with HTP-3 mutants that block HTP-1/2 binding. I realize these mutants may not be in hand but what I am getting at is to find a situation where the role of the HIM-3 non-phospho protein is uncovered. This would lend support to the importance of the phosphorylation as well as the redundant nature of defining chromosome domains.

We have combined *him-3* and *syp-1* nonphosphorylatable mutants, and found no synergy in embryonic inviability (i.e., *syp-1(T452A)* was epistatic); this is shown in Sup.Fig. 14B. We also attempted to analyze *htp-3* mutants in combination with *him-3* phosphomutants as well after requesting the *htp-3(2GK)* strain from the Kim lab, but could not construct the double-mutant strain despite repeated attempts as we described in the response of reviewer #1.

5. Phospho-mimetic mutants: It is surprising that the phospho-mimetic mutants have a more severe phenotype compared to the non-phosphorylated mutants – I would like to see this discussed more and acknowledged that mutation to E or D may not completely mimic the effect of phosphorylation. Are these mutants dominant?

We have added this caveat to the discussion (lines 600–605). While it is true that the phenotypes may arise in whole or part from an unexpected neomorphism introduced by the D or E residue, it seems equally plausible that the S282D/E phenotypes are the same as what a truly constitutively phosphorylated HIM-3 would be. We therefore feel the dominance experiment may not shed much light on this question, and we have opted to prioritize several other of the reviewer-suggested experiments.

6. Please add p values to Figures where appropriate, e.g., Supplemental Figure 2 and 7B.

We have now added p values to all figures and/or figure legends.

***Reviewer #3***:

In this paper Sato-Carlton et al. investigate the post-translational modifications in the meiotic axis HORMA-domain protein HIM-3 and the effects SC restructuring in preparation for the meiotic division. They discovered this modification through Mass Spectrometry and have used a combination of genetics and immunohistochemistry to tease out its role during meiosis. The topic of the study is certainly of interest to the genetics community as post-translational modifications appear to be critical regulators of meiotic processes and our understanding of these is still in its infancy. While the authors present convincing evidence that the phorphorylation of HIM-3 is downstream of SYP-1 and helps in the partitioning of the long and short axes, ultimately, the modification is not essential for the restructuring as these are resolved by the -1 oocytes. Thus, the post-translational modification appears to be adding to the robustness of this process. In this regard, the authors do an excellent job interrogating different models of partitioning and the interplay between the SC proteins and HIM-3 and HTP-1/2. The paper is lacking in two areas: the major phenotypes are in late meiotic events, and while they allude to early defects in meiosis (such as in the syp and plk-2 mutants), the images to evaluate early meiotic events are not provided; and perhaps more importantly, they were unable to identify the relevant kinase—although they only tested a few possible candidates. Thus, while a thorough analysis of new modification, one is left somewhat wanting for more (perhaps an RNAi of known kinases).

While identification of the kinase (or a phosphatase) would tie the paper up in bow, there are a few other issues that need to be clarified prior to publication.

Major Issues:

Figure 1C should have statistical analysis provided. This will show whether the control with Ala is really not significant, as well as to see whether the phosphorylation of S279 is significantly different or not.

We have carried out the significance tests for the FP assay and report this in the Figure legend and the main text. Both S282A and Y279phos peptides show significantly different binding kinetics.

One of the problems in analyzing some of the figures is that we see only snapshots of one or two nuclei and not the whole region or the whole meiotic germ line. This is especially true for Figure 4 (spo-11) but also to address their comments that the timing of SC formation is “normal” where the images are not shown to support this statement. These should be provided in the supplemental data.

Thanks for the suggestion — we have provided enlarged area images for *spo-11* (**Figure 4**) in **Sup. Fig. 6**, shown SC formation in **Sup. Fig. 7**, and SC removal in **Sup. Fig. 9**.

In Supplemental Figure 1C, HIM-3phospho staining appears more robust in the atm-1;atr-1 double mutants. This is not addressed.

We have re-examined our images of *him-3(S277A)* single v.s. *him-3(S277A); atm-1; atl-1* triple mutants. Since mitotic and meiotic problems are quite severe in the background of *atm-1; atl-1* and most of meiocytes appear to carry polyploidy or micronuclei, we failed to quantitatively determine if HIM-3phos signals are more intense in the *him-3(S277A); atm-1; atl-1* mutants compared to *him-3(S277A*) single mutant. Just by observing many gonads from both genotypes, we did not have an impression that HIM-3phos signals are more intense in *him-3(S277A); atm-1; atl-1* triple mutants but rather quite variable depending on the gonad in this mutant. We replaced images with more representative staining to forestall the impression that HIM-3phos is more intense in the *him-3(S277A); atm-1; atl-1* mutant.

P 13 states “In all of the him-3 mutants, HIM-3 proteins localize to the SC at normal levels by pan-HIM-3 antibody staining” but this is not shown. Also for the next sentences: “In him-3 phosphomimetic mutants, HTP-1 was detected on the SC from the leptotene/zygotene transition zone,and SC central elements were polymerized normally.” This is also not shown.

We now show pan-HIM-3 staining in the HIM-3 phosphomutants in **Sup. Fig. 7A**, and HTP-1 staining in **Sup. Fig. 7B**.

Since the HIM-3phos antibody recognizes the combination of Ser 277 and Ser 282, is it possible that the remaining signal on the long arm represents a partially phosphorylated intermediate (on one of the two sites)?

To distinguish Ser277phos and Ser282phos, we have immunostained S277A or S282A single mutants using HIM-3phos antibodies. HIM-3phos antibodies stained the SC in both S277A single and S282A single mutants, indicating that (1) they are a mixture of antibodies recognizing Ser277phos and Ser282phos, and (2) that Ser277 phosphorylation and Ser282 phosphorylation occurs independently. In line with the reviewer’s supposition, we found that the S282A mutation leads to persistence of Ser277phos staining on long arms until -2 diakinesis, while in the S277A mutant, Ser282phos signals become completely confined to short arms by the end of pachytene — the implication being that the remaining long arm signal in the wild type is likely S277phos. We have added this observation in the main text as well as Supplemental Figures 2 and 3. Please see also the detailed response to the same issue raised by Reviewer 1’s major point #2.

P 10 and Figure 3A, they say that there is an enrichment on HIM-3 at the “very end of late pachytene, but the images are not those immediately adjacent to diplotene. This should be quantified as the number of rows before the diplotene transition or by the percent of distance along the leptotene- diplotene germ line. This also comes up on p. 11 in discussion of the HIM-3phos staining in spo-11(me44). I think the term “very late pachytene” needs to be defined since clearly there are still adjacent nuclei that are in pachytene and not diplotene.

We have added the “the last 5 rows of meiocytes” to describe where HIM-3phos becomes enriched at the end of the pachytene zone (line 217).

It would be interesting if the authors say in which stage they observe the enrichment of SYP-1 phosphorylated along the entire length of chromosomes harboring COSA-1 foci in spo-11(me44), because if happens in late stages this conflicts with previous results where a desynapsis is observed in nuclei with subthreshold of CO.

From the current manuscript and our previous work we have found that in wild-type nuclei, short arm enrichment of phospho-SYP-1 is the first, or fastest, differential modification that occurs. Phospho-HIM-3 enrichment appears to come next, followed lastly by asymmetric disassembly of SYPs. To highlight the order of events, we added SYP-1 staining in Fig 2B panels and added the image of HIM-3phos and SYP-1 staining in **Supplemental Figure 1D** to show phospho-HIM-3 enrichment preceding to SYP-1 disassembly. In DSB-sub-threshold nuclei, phospho-SYP-1 is still the fastest, becoming enriched on whole chromosomes that contain CO intermediates before desynapsis (before there is detectable enrichment of pan-SYP-1 on those chromosomes, or loss of pan-SYP-1 from chromosomes that lack CO intermediates) (Fig 4 and 5). Also, since enrichment of SYP-1-phos and desynapsis are events that occur on different chromosomes, we do not think there would be any conflict even if the timing were the same.

The persistence of HTP-1 on both arms of the bivalent in the him-3 phosphomimetic backgrounds is discussed as a potential “timer” for dissociation. However, it is also possible that there is an overall delay in the events of late pachytene- diakinesis. This could at least be addressed by staining for meiotic maturation markers (e.g. wee-1; oma-1; etc) and making sure they appear with the correct timing.

We have carried out this experiment with anti-OMA-2 immunostaining, and found that the timing of progression from late pachytene-diakinesis is normal (**Sup. Fig. 10**).

Minor issues with Figures

Figure 2B does not say that the diakinesis oocyte is the -1 oocyte. Given the discussion of the localization of HIM-3phos in the -1 oocyte, they really should provide images of -1 to -7 oocytes showing the transition.

We have added images of these stages of oogenesis (DAPI, panHIM-3, and HIM-3phos staining) as **Sup. Fig. 1C**.

In the Supplemental Figure 3A, it would be helpful to show again the WT controls.

We have now added an N2 control to this figure (now **Sup. Fig. 4D**), along with a simultaneous depletion of GSP-1 and GSP-2 using *gsp-1::degron; gsp-2; degron* strain as well.

In the Supplemental Figure 3B where they show that HIM-3phos staining was absent from unsynapsed chromosomes, the nuclei they decided to point out with the arrowheads are not the most obvious (at least not the one on the left). I would suggest selecting others to point to since there are multiple better examples (dead center, e.g.) that would make the point more strongly.

Thank you for the suggestion. We have now changed the location of the arrowheads, as suggested (current **Sup. Fig. 5A**).

Figure 5C and Figure 6C, Y axes are not labeled

We have added the label “Fraction of nuclei” to the Y axes.

Referencing:

In the first Figure the authors conclude that the examination of the crystal structure led them to predict that phosphorylation at Ser282 would interfere with HTP-1/2 binding. Kim et al., 2014 needs to be referenced here.

This reference has been added to the text.

The interpretation on p. 12 that recombination intermediates alter the SC in cis was first shown in Machovina et al., 2016 and should be referenced.

This reference has been added with context (lines 337–342).

References for the function of BRC-1 from Janisiw et al 2018 and Li, Saito et al., 2018 should be added on p. 16 when this gene is introduced for its molecular function.

We have now added both of these citations to the text.

Minor edits:

The paper is extremely well written and a delight to read. The only sentence that was confusing was the top of p. 14 starting: “This implies that although HTP-1 may bind…”. I really have no idea what they are trying to communicate here: are they trying to say that there are 2 pools of HTP-1, one that binds phosphorylated HIM-3 and one that binds unphosphorylated and that these much be mutually exclusive to explain the decrease staining of HTP-1 in the HIM-3D or E background?

Thanks for pointing out the confusing construction here. We intended to say that while *in vitro* data has shown it is possible for HTP-1 to bind the closure motifs of both HTP-3 and HIM-3, the drop in HTP-1 intensity upon HIM-3 mutation indicates that HTP-1 does indeed bind to HIM-3 *in vivo*. We have altered the text (around line 422) to provide a hopefully clearer explanation.

• P12, paragraph starting “previous studies showed…” should reference those previous studies.

Thanks for noticing this omission; we have now properly cited the papers this paragraph refers to.

• In legends for Figures 2A, 4C , 6C, Suppl Figs 4C, 7C: capitalize first word.

• Scalebars should be “Scale bars”

• GFP-COSA-1 should be GFP::COSA-1

• Suppl. Fig 7B, left word should read “damage” not “damages”

These changes have now all been fixed. Thanks for pointing out our oversight.

Methods:

Rather than referencing previous papers, please detail the methods for immunostaining, mass spectrometry, and fluorescence polarization (and define Scrum and Eurofin).

We have added these details to the Methods section, and added links to each company such as Eurofins in Sup table 2.

Describe how GFP-1::COSA-1 is visualized (antibody?)

We have now added details on anti-GFP immunofluorescence to the methods section.
