# Supplementary material for: Phosphoregulation of HORMA domain protein HIM-3 promotes asymmetric synaptonemal complex disassembly in meiotic prophase in Caenorhabditis elegans
Source: PLoS Genet. 2020 Nov 11;16(11):e1008968. doi: 10.1371/journal.pgen.1008968 (PMC7717579; doi:10.1371/journal.pgen.1008968)
Supplement: Attachment — Submitted filename: ReResponse to editor.docx [file pgen.1008968.s020.docx]

Response to editor’s and reviewers’ comments, 2^nd^ resubmission, for Sato-Carlton et al., “Phosphoregulation of HORMA domain protein HIM-3 promotes asymmetric synaptonemal complex disassembly in meiotic prophase in *C. elegans*”.

<Editor/Reviewer text in *italics*, our response follows in **bold**:>

*There are few text edits suggested by reviewer 2 and 3 (see below) and I have few small edit suggestions indicated here:*

*Line 109: put a dot after [16-18]*

**Done.**

*Line 320: please insert a sentence telling the reader who is CHK-2*

**This has been added now.**

*Ref 37 is now published and can replace the bioRxiv citation*

**Ref. 36 has now been cited as the PLOS Biology version (Ref 37 was already cited as the eLife version of the earlier bioRxiv citation.)**

Page 10-11: Figure 4C is discussed before 4B, so they should be switched

**This has now been changed (in text and Figure 4)**

Page 12: Figure 5C is discussed before 5B, so they should be switched

**Thank you for the suggestion. For layout reasons, we needed to construct Figure 5 so that panel C contains quantitation of microscopy data shown in A and B; we therefore call out (5A,C) then (5B,C) in order. If we were to place the 5A images and 5A quantification graph, then 5B images and 5B quantification graph in this order from top to bottom, we would need to shrink the images to accommodate two separate graphs. However, we would like to maintain the current size and aspect ratio of our panels for clarity.  We do not anticipate any confusion here, and feel that rearranging the figure and text at this point for strict adherence to a style guideline would instead sacrifice simplicity and clarity, and so prefer to keep it the way it is. We have modified the text to clarify that quantification of Figure 5A images is presented in 5C.**

Figure 1B: consider indicating the closer motifs of HTP-1/2 by a box the same way it’s done for HTP-3 and HIM-3

**Thanks for the suggestion. We had considered this, but since no proteins are known to bind the HTP-1/2 closure motifs (the roles of the HTP-1/2 closure motifs are not known, nor do we discuss them), highlighting them might engender confusion.**

Figure 4 and 5 and 6D: on my computer it’s hard to see the magenta over the white background, consider moving the labels out of the panel to the top of each panel

**Figures 4,5,6: The suggested change has been made.**

Sup figure 5C, 7A, 13B, 14B: instead N2 please put “N2 wild type” or “wild type”

**5C,7A,13B,14B: changed.**

Figure Sup 7B: the word “Merged” is missing on some of the panels

**We have added a “Merged” label in the last column of panels.**

Sup figure 9, 10, 13, 14C: instead control indicate/only indicate the specific genotype used

**Done: S9, S10, 13, 14C**

Reviewer's Responses to Questions

**Comments to the Authors:**

**Please note here if the review is uploaded as an attachment.**

Reviewer #1: The authors have added a substantial amount of new data, which addressed some of the previous concerns. Especially, the distinct behavior of HIM-3 phosphorylation at S277 vs. S282 is intriguing, and identification of the kinases/phosphatases that are responsible for HIM-3 phosphorylation/dephosphorylation in synapsis- and crossover-dependent manners will be important topics for future research. Although the authors were not able to test the redundancy for establishing the asymmetric localization of HTP-1/2 due to technical difficulties, the additional experiment has placed HIM-3 phosphorylation downstream of PLK-2 and SYP-1 phosphorylation, which is a significant addition. Finally, the model presented in the supplemental figure is informative, and the revised Discussion adequately clarifies and explains the differential phenotypes in phospho-defective vs. phospho-mimetic mutants.

Overall, this study is well executed and will be of interest to the meiosis community. I support the publication of this work.

Reviewer #2: The revised manuscript by Sato-Carlton et al., describes the identification and functional characterization of HORMA axis protein, HIM-3 phosphorylation, in C. elegans meiosis. The authors have done a very good job addressing the previous reviews. I found a couple of typos:

1. Line 226 – please remove the first “single”

2. Line 516 – “remains” should be “remain”

**These two changes have both been made now.**

Reviewer #3: The revised manuscript of Carlton et al addresses most of the comments of all of the reviewers and importantly adds statistics, new supplemental data, and important controls about the specificity of their antibody. While they are still lacking insight into the kinases and phosphatases that are controlling this modification, the manuscript as is highlights important biology that raises new areas for investigation that will be relevant to the field as a whole.

Three minor issues should be addressed for publication:

p. 1 last line: “…division of the chromosome into two segments or arms by..” I had to read this 3 times ot parse if correctly perhaps: “division of the chromosome into segments (or “arms”) by…”

**We have tried to correct this line to be more clear, as follows: “The best-understood of these is found in the nematode *Caenorhabditis elegans*: after the single off-center crossover divides the chromosome into two segments, or arms, several chromosome-associated proteins or post-translational modifications become specifically partitioned to either the shorter or longer arm, where they promote the correct timing of cohesion loss through as-yet unknown mechanisms.”**

Line 82: Although the prevailing dogma is that chromosomes receive ”one and only one crossover” there is actually genetics data from Hodgkin et al. (1979), that rare DCOs do occur. In males, Zetka and Rose reported DCOs in wild type and rec-1 mutants. Lim et al 2008 showed that there can be up to 3% DCOs on Chr III in hermaphrodites and 1% in males using molecular markers spanning >96% of the genome. Tsai et al 2008 found DCOs on chr I using cytological markers. So, using these data together and extrapolating to the whole genome, while most receive only a single crossover, telomere-proximal crossovers likely do happen in up to 15% of meioses on at least 1 chromosome.

**The reviewer is correct that we oversimplified here; we have changed the line to read “each chromosome normally receives a single crossover” to be consistent with these reports.**

Line 420-425: Since the table shows a statistically significant loss of viability in the Htp-1::flag; him-3(S282E) mutant, this needs to at least be addressed in the text and not only in the response to the reviewers.

**We have now added this point in the main text.**
